# Serial High-Sensitivity Troponin T in Post-Primary Angioplasty Exercise Test

**DOI:** 10.5935/abc.20160029

**Published:** 2016-04

**Authors:** Humberto Andres Vaz, Ana Paula Vanz, Iran Castro

**Affiliations:** Instituto de Cardiologia - Fundação Universitária de Cardiologia, Porto Alegre, RS - Brazil

**Keywords:** Troponin T, Ischemia, Myocardial Infarction, Exercise Test, Angioplasty

## Abstract

**Background:**

The kinetics of high-sensitivity troponin T (hscTnT) release should be studied in different situations, including functional tests with transient ischemic abnormalities.

**Objective:**

To evaluate the release of hscTnT by serial measurements after exercise testing (ET), and to correlate hscTnT elevations with abnormalities suggestive of ischemia.

**Methods:**

Patients with acute ST-segment elevation myocardial infarction (STEMI) undergoing primary angioplasty were referred for ET 3 months after infarction. Blood samples were collected to measure basal hscTnT immediately before (TnT_0h_), 2 (TnT_2h_), 5 (TnT_5h_), and 8 hours (TnT_8h_) after ET. The outcomes were peak hscTnT, TnT_5h_/TnT_0h_ ratio, and the area under the blood concentration-time curve (AUC) for hscTnT levels. Log-transformation was performed on hscTnT values, and comparisons were assessed with the geometric mean ratio, along with their 95% confidence intervals. Statistical significance was assessed by analysis of covariance with no adjustment, and then, adjusted for TnT_0h_, age and sex, followed by additional variables (metabolic equivalents, maximum heart rate achieved, anterior wall STEMI, and creatinine clearance).

**Results:**

This study included 95 patients. The highest geometric means were observed at 5 hours (TnT_5h_). After adjustments, peak hscTnT, TnT_5h_/TnT_0h_ and AUC were 59% (p = 0.002), 59% (p = 0.003) and 45% (p = 0.003) higher, respectively, in patients with an abnormal ET as compared to those with normal tests.

**Conclusion:**

Higher elevations of hscTnT may occur after an abnormal ET as compared to a normal ET in patients with STEMI.

## Introduction

Cardiac troponins (cTn) are highly sensitive and specific biomarkers for the detection of myocardial necrosis. They are an essential complement to the clinical and electrocardiographic criteria for the diagnosis of acute myocardial infarction (AMI) according to the guidelines developed by the *European Society of Cardiology* (ESC), *American College of Chest Physicians* (ACCF), *American Heart Association* (AHA) and *World Heart Federation* (WHF).^1^ The cTn not only added agility to diagnostic confirmation,^2,3^ but proved to be very useful in choosing between different therapeutic strategies^4-9^ and in identifying patients at higher risk for future cardiovascular events.^10,11^

Recent advances have yielded greater accuracy for those tests. They are now called high-sensitivity troponins, because they have the ability to be detected at small concentrations with higher accuracy, including in individuals apparently free from cardiovascular disease.^2^ Consequently, their kinetics has been the focus of several studies in cardiology. One important topic is their elevation in transient ischemia during physical or pharmacological stress tests.^12-16^ The present study was aimed at assessing the kinetics of high-sensitivity troponin T (hscTnT) by use of serial measurements after an exercise test (ET) performed in ST-segment elevation myocardial infarction (STEMI) patients, and at comparing the changes in that biomarker levels on abnormal *versus* normal tests.

## Methods

Cross-sectional study performed from December 2010 to August 2012 at the Institute of Cardiology/Fundação Universitária de Cardiologia (IC/FUC), Rio Grande do Sul state, Brazil. The inclusion criteria were patients aged at least 18 years, diagnosed with STEMI, undergoing anticoagulant therapy and adjuvant antiplatelet therapy during follow-up at a coronary care unit, according to the ACCF/AHA guideline for the management of STEMI, 2013,^17^ and primary angioplasty with conventional stents with the following angiographic conditions: final TIMI III flow in the affected vessel and complete revascularization, defined as no stenosis ≥ 50% in another epicardial coronary artery. The exclusion criteria were as follows: patients without full conditions to exercise on a treadmill and presence of left bundle-branch block or left ventricular overload with ST-segment depression ≥ 1 mm on baseline electrocardiogram. The presence of lesions in the left main coronary artery or equivalent, unstable clinical findings, planned coronary artery bypass grafting, and impossibility to follow the research protocol and/or refusal to participate in the study were also considered exclusion criteria.

Of the 104 patients recruited, 9 did not undergo initial assessment, one underwent coronary artery bypass grafting, 7 withdraw the study before undergoing ET, and one could not exercise on a treadmill due to orthopedic problems, leaving 95 participants to be included in this study sample.

The following data were collected: anthropometric data; laboratory data; medical history; and relevant data on primary angioplasty and coronary angiography. Patients were invited to participate in the study before hospital discharge. When eligible, they provided written informed consent (WIC) after being instructed on the ET and the research protocol.

Exercise testing was recommended 3 months after STEMI, but, because of logistic factors and issues related to scheduling and participants' displacement, that period varied, the median being 108 days (interquartile interval: 93-145). The blood collections for hscTnT measurement were as follows: immediately before the ET, and after 2 hours (mean, 2.7 ± 0.6 hours), 5 hours (mean, 5 ± 0.6 hours) and 8 hours (mean, 8.6 ± 0.6 hours).

The study was approved by the Research Ethics Committee (protocol nº 4391/09) and abided by the Helsinki declaration. All participants provided the WIC before undergoing any intervention.

### Exercise test protocol

The stress test adopted was the symptom-limited ET on treadmill according to the Bruce protocol.^18^ It was scheduled within approximately 3 months after the STEMI, maintaining the complete treatment, including beta-blockers and nitrates. Valid tests were those with 12-lead electrocardiographic tracing in the sitting and standing up position, at rest and during exercise, with stable baseline and no interferences.

Blood pressure and continuous heart rate measurements were taken, and the maximum load was calculated in METS. The ET would be immediately interrupted in case of sustained ventricular tachycardia, blood pressure drop during exertion, ST-segment depression ≥ 2mm and progressive chest pain during the procedure. The ET was conducted by a cardiologist with no knowledge on baseline hscTnT (TnT_0h_) or any of the following measurements. The abnormality criteria considered for the ET were: on electrocardiogram, horizontal/descending ST depression ≥ 1mm at 0.08s after the J point and complex ventricular arrhythmias; and symptoms or clinical findings characteristic of myocardial ischemia during exertion.

### hscTnT collections

Peripheral blood samples were obtained according to the manufacturer's instructions. They were collected before the ET (TnT_0h_), and after 2 hours (TnT_2h_), 5 hours (TnT_5h_) and 8 hours (TnT_8h_). All patients had a meal before the baseline collection and ET, and remained on the hospital premises with no physical activity until the next venous puncture. Blood was collected at the same place of exercise testing. To ensure rest, the participants remained sitting for 30 minutes before the collection. The blood samples were always processed by the same professional immediately after collection. Troponin T STAT (Short Turn Around Time) assay was analyzed by using the commercially available Elecsys 2010 analyzer (Roche Diagnostics, batches nº 153401, 157120, 160197, 163704), which uses the chemiluminescence method (analysis of two monoclonal antibodies specifically directed against human troponin T). The limits of the blank, of detection and maximum are 3ng/L, 5ng/L and 10,000ng/L, respectively. The limit of test quantification was 13ng/L (functional sensitivity), corresponding to the lowest concentration that can be measured in a reproducible way with coefficient of variation (CV) ≤ 10%. The 99th percentile detected in a reference population was 14ng/L.^19^ The information for calibration of each assay is specifically established according to each batch used. Each batch was adapted to the analyzer by using the Elecsys Troponin T STAT CalSet calibrator no later than 24 hours after registering the reagent kit. New calibrations were performed as needed, according to the manufacturer.

### Statistical analysis

The normal distribution of the continuous variables in this sample was assessed by using the Kolmogorov-Smirnov test. The continuous variables were presented as mean and standard deviation, and, in case of asymmetrical distribution, as median (interquartile interval: p25-p75). Categorical variables were presented as absolute count and percentages. To compare between different categories of hscTnT changes and the presence of normal or abnormal ET, Fisher exact test was used. To compare hscTnT values between two groups, Mann-Whitney U test was used. In addition, Spearman coefficient was adopted to assess the correlation between age and hscTnT values, and between creatinine clearance and hscTnT values. Due to the asymmetrical distribution of hscTnT values, logarithmic transformation was used. To assess hscTnT changes between the groups with normal and abnormal ET, the following outcomes were used: post-ET peak troponin (peak TnT); ratio between troponins collected in the fifth hour and at baseline (TnT_5h_/TnT_0h_); and area under the blood concentration-time curve. Due to the logarithmic transformation, the hscTnT values were presented as geometric means, and the comparisons between the groups were summarized by using the geometric mean ratio with their respective confidence intervals. The statistical significance of those comparisons was assessed in a model of analysis of covariance (ANCOVA), initially without adjustments, and then adjusting for TnT_0h_, age, sex and additional variables (METS, percentage of the maximum heart rate achieved, anterior left ventricular wall STEMI, and creatinine clearance estimated with the *Cockcroft-Gault* formula). A p value <0.05 was considered statistically significant, and the entire analysis was elaborated by using the Statistical Package for the Social Sciences (SPSS) software, version 21.0 (SPSS Inc., Chicago, IL, USA).

## Results

This study included 95 patients diagnosed with STEMI, treated with primary angioplasty and submitted to ET 3 months after the initial event. The mean age of this sample was 54.25± 11 years, with a higher prevalence of the male sex (81%).

The right coronary artery was affected in 46% of the cases, followed by the anterior descending (43%) and the circumflex (8%) arteries. On coronary angiography, three-vessel disease was detected in only 4% of the patients, no lesion being detected in the left main coronary artery. Only 5% of the patients underwent stent implantation in a second epicardial vessel, right after treating the affected coronary artery. Whenever anatomically possible, the patients underwent complete revascularization, defined as residual lesions smaller than 50%. Table 1 shows the baseline characteristics of this sample.

**Table 1:** Characteristics of the patients according to abnormal or normal exercise tests (ET).

**Characteristics**	**abnormal ET (n=13)**	**normal ET (n=82)**
**Anthropometric**		
Age (years)	59±12	54±10
Male sex n (%)	12 (92)	65 (79)
BMI (kg/m^2^)	28±3	27±5
**Risk factors (%)**		
Current or previous smoking	9 (70)	53 (65)
Systemic arterial hypertension	9 (70)	65 (80)
Family history of CAD	2 (15)	30 (37)
Diabetes	1 (8)	13 (15)
Dyslipidemia	7 (54)	62 (76)
**Acute myocardial infarction**		
Time until treatment (hours)	5 (2 - 6)	4 (2 - 5)
Anterior wall location n (%)	3 (23)	44 (53)
**Number of vessels affected n (%)**		
1	8 (61)	64 (78)
≥2	5 (38)	18 (22)
**Stents n (%)**		
1	9 (70)	68 (80)
2	4 (30)	14 (17)
1st stent length (mm)	27±8	21±7
2nd stent length (mm)	19±10	18±8
**ET: additional parameters**		
% of maximum HR achieved	79±12	80±12
Double product (x10^3^)	22±5	22±6
METS	8±2	8±2
**Biochemistry**		
Creatinine clearance (mL/min)	90±25	100±30
TnT_0h_	6.7 (4.7 - 7.4)	5.4 (3 - 9.5)

Data presented as mean ± standard deviation (SD), n (percentages) and median (p25 - p75); ET: exercise test; BMI: body mass index; CAD: coronary artery disease; HR: heart rate; METS: metabolic equivalents; TnT_0h_: baseline troponin T.

Regarding medication, most patients used a combination of acetylsalicylic acid (97%), clopidogrel (92%), statins (96%), beta-blockers (92%) and angiotensin-converting-enzyme inhibitors (94%) at the time of the ET. A smaller proportion (4%) of patients used oral or sublingual nitrates to relieve anginal symptoms before the ET.

Of the total sample, 13 patients were classified as having an abnormal ET. Of those, 11 (84%) had persistent ST-segment depression ≥ 1 mm during the test, one patient (8%) had non-sustained ventricular tachycardia associated with clinical signs of coronary artery disease (CAD), and another (8%) had progressive angina *pectoris*, requiring the interruption of the procedure.

The values of creatinine clearance and ET performance were similar in the groups with normal and abnormal ET. The frequencies of the traditional risk factors for CAD and of anterior left ventricular wall STEMI were similar in both groups. There was a trend towards the use of longer stents (p=0.06) in the group with abnormal ET as compared to that with normal ET.

In 35 (37%) patients, TnT_0h_ was undetectable. Smoking (p = 0.03) and age (p < 0.001), directly, and creatinine clearance (p < 0.01), indirectly, were associated with higher. TnT_0h_ values. Ten patients (19%) reached or exceeded the clinical decision level (14 ng/L), and that finding was more frequent in the abnormal ET than in the normal ET group, 46.2% *versus* 14.6%, respectively (p = 0.015).

Higher hscTnT geometric means were identified at the time of the third collection (TnT_5h_) in patients with abnormal ET than in those with normal ET, as well as a decrease in those values in the fourth collection (TnT_8h_). The ANCOVA showed a 71% greater peak TnT in patients with abnormal ET as compared with those with normal tests, 54% greater with adjustments for TnT_0h_, sex and age (p = 0.003), and 59% greater after adjustment for additional factors (p = 0.002), as shown in Table 2.

**Table 2:** Non-adjusted and adjusted comparisons between the groups with abnormal exercise test (ET) *versus* normal ET for selected outcomes.

			**Non-adjusted analysis**		**Adjusted analysis for TnT_0h_, age and sex**		**Analysis with additional adjustment†**	
**Outcome**	Abnormal ET* (n = 13)	Normal ET* (n = 82)	**geometric mean ratio (95% CI)**	**p**	**geometric mean ratio (95% CI)**	**p**	**geometric mean ratio (95% CI)**	**p**
Primary								
peak TnT (ng/L)	13.15	7.69	1.71 (1.07 - 2.73)	0.025	1.54 (1.14 - 2.07)	0.003	1.59 (1.17 - 2.15)	0.002
Secondary								
TnT_5h_/TnT_0h_	1.90	1.22	1.56 (1.16 - 2.10)	0.004	1.51 (1.11 - 2.04)	0.008	1.59 (1.17 - 2.15)	0.003
AUC (ng/L)^2^	84.3	55.0	1.54 (0.98 - 2.39)	0.058	1.39 (1.10 - 1.77)	0.007	1.45 (1.14 - 1.85)	0.003

*Data are presented as geometric means; CI: confidence interval; p: statistical significance; ET: treadmill exercise test; TnT: high-sensitivity troponin T; AUC: area under the curve;

† additional adjustment for baseline troponin T (TnT_0h_), metabolic equivalent, percentage of maximum heart rate reached, anterior wall infarction and creatinine clearance (Cockcroft-Gault method).

**Figure 1:**
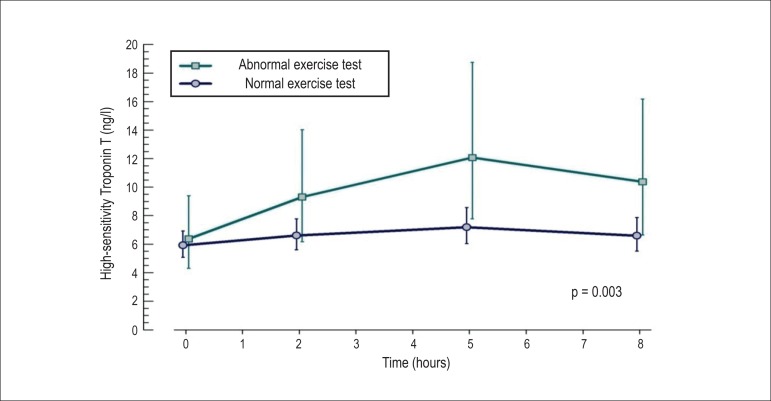
Variation of hscTnT over time in the groups of normal and abnormal exercise tests, presenting the geometrical means, their respective confidence intervals and significance for the area under the curve analysis (AUC).

When comparing the groups with normal and abnormal ET, the analysis of the area under the blood concentration-time curve of the hscTnT values showed statistical significance (p = 0.003) after adjustments (Figure 1).

## Discussion

Using a high-sensitivity troponin T assay, we demonstrated that elevations in that marker, adjusted for the baseline levels, are greater in ET with changes suggestive of transient ischemia as compared to normal tests of STEMI patients treated with primary angioplasty. The ET changes defined in this study were associated with hscTnT increments, especially after the fifth hour, even with adjustments for additional variables, such as load, percentage of maximum heart rate achieved and creatinine clearance. The cTn released into blood stream seems to originate initially from the cytosol content, and later from the cardiomyocyte structural content. The latter would account for the sustained curve of cTn known in AMI, and would translate an irreversible injury to the sarcomere proteins. That difference is the basis for the questions related to the transient troponin increase in the absence of myocardial necrosis.^20^

Hessel et al., in a study inducing cardiomyocytes to metabolic inhibition, have concluded that the release of troponin T (cTnT) and I (cTnI), in their both intact and degradation product forms, occurs simultaneously and only after necrosis.^21^ However, hypothetical mechanisms for the transient release are as follows: apoptosis;^22^ normal cardiomyocyte turnover;^23^ passage of degradation fragments through the intact cell membrane;^24^ and formation and passage of vesicles with cytosol content to the extracellular space.^20^

In previous studies with fourth-generation cTn assays performed after stress testing, the results remained undetectable, below the CV limit of 10%, or not associated with ischemia induction.^25-29^ Another study using high-sensitivity cTnI (hscTnI), however, has found changes proportional to the intensity of the ischemia (mild and moderate-to-severe) estimated on myocardial perfusion imaging, when the sample was collected 2 and 4 hours after the stress test. In that same study, changes in troponin levels in patients with different ischemic categories were indistinguishable using conventional troponin assays.^12^

The present study assessed a specific population of patients with a sequela of STEMI, and measuring those markers at baseline and after ET in individuals with structural heart disease is particularly important. In a previous study with 118 patients, measuring cTnI before Bruce protocol symptom-limited ET, and then 8-12 and 24 hours after, no correlation of the elevation in biomarker levels with the presence of multiarterial disease and ET changes was found. However, on multivariate analysis, ejection fraction ≤ 50% was an independent variable for cTnI elevations above the 99th percentile.^30^ Another study has assessed serial hscTnT and hscTnI after myocardial perfusion imaging stress testing, and none of those markers could identify patients with reversible ischemia. A significant increase in hscTnI was comparable to hscTnT levels at all collection times in the presence of previous AMI, but without reversible ischemia (p < 0.001 *versus* baseline collection). The baseline cTn concentrations in that study seemed to be influenced by variables related to myocardial structural changes.^13^ Another study using hscTnT after magnetic resonance imaging has detected small amounts 1 and 3 hours after non-pharmacological stress, not fulfilling criteria for AMI, but the levels were related to the intensity of the ischemia found. History of diabetes, CAD, lower creatinine clearance and ejection fraction were more frequently found in patients with moderate-to-severe ischemia.^16^ Other studies demonstrating cTnI release in individuals with heart failure^31^ submitted to exercise or in marathon runners with exercise-induced high blood pressure^32^ could also indicate a role for the presence of those markers in different left ventricular overload situations.

In the present study, a late ET was performed 3 months after AMI to avoid the detection of TnT_0h_ levels in the descending curve because of the primary tissue injury caused by AMI. Our study found that higher TnT_0h_ levels correlate with smoking, older age and lower creatinine clearance. The last two findings are similar to those studies using hscTnT^33^ and hscTnT and hscTnI.^13^

We believe that the values found were not actually related to new coronary events, because of the small variations and the early descent, but rather to cases with imbalance between oxygen offer and demand, based on the significantly lower levels of high-sensitivity troponins found in that situation.^34^ Lower values found on the initial assessment of patients for acute coronary syndrome seemed not related to type I AMI (ischemia due to atherosclerotic plaque rupture, thrombus formation, fissure and spontaneous dissection),^35^ and regardless of the cause of hscTnT release in circulation, increases in the marker can be related to higher mortality. Data from the SWEDEHEART Registry have shown that patients suspected of having acute coronary syndrome and hscTnT levels greater than 14 ng/L had higher adjusted mortality rates; however, only 18.2% of them actually had had an AMI.^36^ We could infer that, even without knowing the exact mechanism of hscTnT release, increases in that marker, especially from the 99th percentile on, could indicate other changes related to the post-STEMI period, which should, from now on, be studied.

The use of that marker in association with the traditional risk parameters in ET could indicate one more risk criterion. However, this cross-sectional study assessed the kinetics of that marker in a limited population. The meaning of those changes in association with ET should be assessed in large prospective studies. The use of high-sensitivity assays will not often identify patients at risk without high hscTnT levels, above the clinical decision limits, or with small transient changes. The clinical setting should be valued when considering the circumstances under which low hscTnT levels can be detected in circulation.

## Conclusion

Serial hscTnT elevations after ET were demonstrated. In abnormal tests, after determining the baseline values, the hscTnT levels are significantly higher as compared to normal ET in STEMI patients. In transient abnormalities suggestive of myocardial ischemia in ET, hscTnT shows a pattern of elevation followed by an early descent. Higher baseline values are related to smoking, older age and lower creatinine clearance levels. In that population, elevated levels, especially from the 99th percentile, can indicate a higher risk or myocardial structural injury.

### Limitations

Exercise testing without the addition of imaging tests has limitations. Thus, the presence of hscTnT changes cannot be considered a manifestation of residual ischemia. In addition, there were neither a control group nor echocardiographic data to correlate left ventricular structural changes with the hscTnT kinetics. The pathway to the knowledge of the real meaning of those changes regarding the increment of prognostic data should be delineated in prospective studies with a larger number of participants.
